# Multi-Breed Genome-Wide Association Analysis Reveals Candidate Genes for Growth and Body Conformation Traits in Four Populations of Native and Crossbred Chinese Sheep

**DOI:** 10.3390/ani16162605

**Published:** 2026-08-20

**Authors:** Erkinbay Azbergenov, Tao Jiang, Ruizhi Yang, Qifeng Gao, Fuming Kou, Yaxuan Liao, Yang Yang, Shudong Liu

**Affiliations:** 1 College of Life Science and Technology, Tarim University, Alar 843300, China; azbergenoverkinbay@gmail.com (E.A.); forromaways1889@gmail.com (R.Y.); gqfsryd1415926@163.com (Q.G.); 2 Xinjiang Production and Construction Corps Key Laboratory of Tarim Animal Husbandry Science and Technology, Alar 843300, China; jiangtao888888@126.com (T.J.); koufumin63@126.com (F.K.); 18674769403@163.com (Y.L.); 3 Key Laboratory of Livestock and Forage Resource Utilization in the Tarim Basin, Ministry of Agriculture and Rural Affairs, Alar 843300, China; 4 College of Animal Science and Technology, Tarim University, Alar 843300, China; 5 State Key Laboratory of Sheep Genetic Improvement and Healthy Breeding, Xinjiang Academy, Shihezi 832000, China

**Keywords:** genome-wide association study, growth traits, body conformation, multi-breed sheep, skeletal development, candidate genes

## Abstract

Growth and body conformation traits are key determinants of meat production efficiency and economic performance in sheep. However, the genetic architecture underlying these complex traits remains incompletely understood, particularly across multi-breed populations. In this study, we performed a genome-wide association study (GWAS) for seven growth and developmental traits in a combined population of 401 sheep, including Qira Black, Kyrgyz, Dorset × Hu crossbred, and Suffolk × Karakul crossbred sheep. After genotype harmonization and quality control, 47,674 autosomal SNPs were retained for analysis. Population structure was assessed using principal component analysis, and association testing was conducted using a mixed linear model incorporating breed, principal components, and a kinship matrix. A total of 44 independent loci were detected at a nominal significance threshold, encompassing 112 candidate genes. The strongest association was identified for cannon bone circumference near *RPS6KA5* (Chr7; *p* = 1.40 × 10^−7^). Several biologically relevant genes involved in osteogenesis, cartilage development, and metabolic regulation were detected, including *STEAP3*, *SLC26A2*, *PPARGC1B*, *COL11A1*, *CALN1*, and *CITED2*. Two genomic regions exhibited pleiotropic effects, which were identified as being associated with multiple traits, suggesting shared genetic regulation of correlated skeletal characteristics. These findings are consistent with a polygenic architecture underlying growth trait in sheep and highlight candidate genomic regions potentially involved in skeletal development and body conformation. Although further validation is required, the identified loci provide preliminary evidence for regions that may influence growth-related phenotypes and offer a reference for future molecular breeding efforts in indigenous and crossbred sheep populations.

## Simple Summary

Growth and body conformation traits are important determinants of meat production and economic performance in sheep. Understanding their genetic basis can enhance breeding efficiency and support sustainable genetic improvement. In this study, we conducted a genome-wide association study (GWAS) to identify genomic regions associated with seven growth and body conformation traits in 401 sheep from four populations: Qira Black, Kyrgyz, Dorset × Hu crossbred, and Suffolk × Karakul crossbred sheep. After genotype quality control, 47,674 SNP markers were analyzed using a mixed linear model accounting for population structure and genetic relatedness. A total of 44 independent genomic regions were detected at a nominal significance threshold, containing 112 candidate genes potentially involved in skeletal development, cartilage formation, muscle growth, and metabolic regulation.

## 1. Introduction

Small ruminants contribute substantially to global livestock production systems, providing meat, wool, milk, and other products that support economic sustainability and rural livelihoods [1]. Growth and body conformation traits are key determinants of productivity and carcass performance in sheep, and understanding their genetic architecture is essential for improving breeding efficiency and long-term genetic gain. Sheep were domesticated approximately 10,500 years ago in the Fertile Crescent [2], and subsequent artificial and natural selection has led to the development of diverse breeds adapted to distinct environmental conditions [3].

Genome-wide association studies (GWAS) have become a powerful approach for dissecting the genetic basis of complex quantitative traits in livestock [4]. In sheep, GWAS have identified genomic regions associated with growth, body size, and other economically important characteristics [5]. For example, Li et al. [6] detected loci associated with growth traits in Hu sheep using the Ovine SNP40K BeadChip, while Tao et al. [7] identified candidate genes related to muscle differentiation and metabolism in Qira Black sheep using a high-density SNP chip. Tuersuntuoheti et al. [8] further confirmed that body height, body length, and chest girth are quantitative traits controlled by multiple genes, and Zhou et al. [9] identified some genes associated with wool color in Qira Black sheep. In addition, the GWAS method has been applied to investigate other traits in sheep, including horn development [10], reproductive seasonality [11], and environmental adaptation [12].

Qira Black sheep (*Ovis aries*) are a native breed primarily distributed in the arid and semi-arid regions of southern Xinjiang, China, representing an important local genetic resource. Kyrgyz sheep are another native breed from the Kizilsu Kirghiz Autonomous Prefecture in Xinjiang, characterized by strong environmental adaptability and economic relevance in pastoral production systems. In addition, crossbreeding strategies such as Dorset × Hu and Suffolk × Karakul are commonly employed to enhance growth performance and meat production traits. Investigating growth-related genetic variation across these indigenous and crossbred populations provides an opportunity to evaluate shared and population-specific association signals.

Despite these advances, most previous studies have focused on single-breed populations and a limited number of phenotypes, which restricts a comprehensive understanding of the genetic architecture underlying growth and development traits. The extent to which associated loci are consistent across diverse genetic backgrounds, including indigenous and crossbred populations, remains insufficiently explored. Furthermore, integrative analyses that combine SNP array genotypes with whole-genome sequencing (WGS)-derived variants are still relatively limited in sheep, potentially constraining mapping resolution and cross-population validation of association signals.

Therefore, the present study aimed to perform a multi-breed genome-wide association analysis of seven growth and body conformation traits in Qira Black, Kyrgyz, Dorset × Hu crossbred, and Suffolk × Karakul crossbred sheep by integrating Illumina Ovine SNP50K BeadChip and whole-genome sequencing datasets. By incorporating multiple populations and harmonizing multi-platform genotype data, this study seeks to improve the characterization of genomic regions associated with growth traits and to enhance understanding of their polygenic architecture across diverse genetic backgrounds.

## 2. Materials and Methods

### 2.1. Phenotypic Data Collection

A total of 186 healthy Qira Black sheep ewes aged 1–3 years were selected from Xinjiang Hetian Qira Jinken Agricultural and Animal Husbandry Science and Technology Co., Ltd. (Hetian, China). All animals were maintained under uniform feeding and management conditions to minimize environmental variation. Individuals were selected based on health status and the availability of complete phenotypic records.

Additionally, 100 Kyrgyz sheep were sampled from Wuqia County, Kizilsu Kirghiz Autonomous Prefecture, Xinjiang, China. These animals were raised under standard local management systems and were selected according to health status and the availability of complete growth trait records.

The hybrid populations consisted of two crossbred groups maintained at the Tianshan Meat-Type Sheep Breeding Center, Wensu County, Aksu Prefecture, Xinjiang, China. The first group included 90 Dorset × Hu crossbred sheep, and the second group comprised 25 Suffolk × Karakul crossbred sheep. These crossbred populations were derived from structured multi-generation breeding programs aimed at improving growth performance and meat production traits.

Only female sheep were included in this study to minimize sex-related phenotypic variation in growth and body conformation traits. All animals included in this study were healthy and had complete phenotypic measurements. Detailed population information is summarized in Table 1.

### 2.2. Genome Reference Harmonization and SNP Identifier Standardization

Qira Black sheep were genotyped using the Illumina Ovine 50K SNP BeadChip (Beijing Compass Biotechnology Co., Ltd. Beijing, China), whereas Kyrgyz sheep and Dorset × Hu and Suffolk × Karakul crossbred sheep were subjected to whole-genome sequencing (WGS). Because the SNP array coordinates and WGS variant calls were originally based on different reference genome assemblies, harmonization was required prior to downstream analyses.

To ensure consistency across datasets, all genotype data were aligned to the Oar_v4 (OviAri4) reference genome assembly. Genomic coordinates of the Qira 50K SNP markers were converted to Oar_v4 using the UCSC Genome Browser LiftOver tool [13]. Only successfully converted loci were retained for subsequent analyses, while unmapped or ambiguously mapped loci were excluded.

To facilitate direct merging of SNP array and WGS datasets, SNP identifiers were standardized using a unified format (chromosome: position). The bim files were modified using PLINK v1.9 [14]. Chromosome coding was specified using the --chr-set 27 parameter to accommodate the ovine autosomal and sex chromosome structure.

### 2.3. Extraction of 50K SNP Positions from WGS Data

Whole-genome sequence variants from Kyrgyz sheep and Dorset × Hu and Suffolk × Karakul crossbred sheep were converted from Variant Call Format (VCF) to PLINK binary format (.bed/.bim/.fam) using PLINK v1.9 [14]. Based on the lifted Oar_v4 coordinates of the Qira Black 50K SNP panel, corresponding loci were extracted from each WGS dataset using the --extract function in PLINK v1.9.

Following coordinate harmonization and locus matching, 49,776 SNPs were retained in the Kyrgyz dataset, and 49,999 SNPs were retained in the combined Dorset × Hu and Suffolk × Karakul crossbred dataset. These results indicate a high level of positional concordance between the SNP array and WGS-derived variants, supporting successful reference genome alignment and marker matching.

### 2.4. Dataset Merging and Quality Control

Following reference genome harmonization and SNP position alignment to Oar_v4 (OviAri4), genotype data from Qira Black sheep (50K SNP array), Kyrgyz sheep (WGS), and Dorset × Hu and Suffolk × Karakul crossbred sheep (WGS) were merged using PLINK v1.9 [14].

During the merging process, 660 multi-allelic variants were identified as allele conflicts across datasets. These loci were removed using the missnp file generated by PLINK v1.9, ensuring that only bi-allelic SNPs were retained. Prior to quality control, the merged dataset comprised 401 individuals and 50,940 SNPs representing the 50K SNP panel loci harmonized across all datasets rather than the union of all WGS-derived variants.

Quality control procedures were conducted using PLINK v1.9. SNPs with a genotype missing rate >5% (--geno 0.05) were excluded. Subsequently, SNPs with a minor allele frequency (MAF) <0.05 (--maf 0.05) were removed to eliminate rare variants with limited statistical power.

Individual-level missingness was assessed using --mind 0.05; no individuals exceeded the 5% threshold, and therefore no samples were excluded. Chromosome coding was specified using --chr-set 27 to accommodate the ovine genome structure.

Hardy–Weinberg equilibrium (HWE) filtering was not applied due to the pronounced population structure and admixture among the four sheep populations, as deviations from HWE may reflect true population stratification rather than genotyping error. After quality control, the final dataset consisted of 401 individuals and 47,674 SNPs.

### 2.5. Population Structure Analysis

Population structure was evaluated using principal component analysis (PCA) implemented in PLINK v1.9 [14]. Prior to PCA, linkage disequilibrium (LD) pruning was performed using a sliding window approach (--indep-pairwise 50 5 0.2) to remove highly correlated SNPs and minimize bias due to extended LD blocks.

PCA was conducted on the LD-pruned dataset using the --pca 20 option, and the first 20 principal components (PCs) were computed. The top three principal components were retained for visualization and interpretation of population stratification. The proportion of variance explained by each component was derived from the corresponding eigenvalues.

PCA plots were generated using R software (R version 4.5.3; R Core Team, 2026) [15] and visualized with the ggplot2 package (v4.0.3) [16].

### 2.6. Genome-Wide Association Analysis

Genome-wide association analysis was performed using a mixed linear model (MLM) implemented in TASSEL (v5.2.96) [17]. Phenotypic data from Qira Black, Kyrgyz, Dorset × Hu crossbred, and Suffolk × Karakul crossbred sheep were merged into a unified dataset containing seven common growth and body conformation traits: body height (BH), body length (BL), chest girth (ChG), chest width (ChW), cannon bone circumference (CBC), hip height (HH), and hip width (HW). Breed information was included as a categorical fixed effect.

The MLM was defined as follows:

*y* = *Xβ* + *Zu* + *e* (1)

where *y* is the vector of phenotypic observations; *X* is the incidence matrix of fixed effects, including SNP genotype, breed, and the first five principal components; *β* is the vector of fixed-effect coefficients; *Z* is the design matrix relating individuals to random effects; *u ~ N*(0, *Kσ_u_*^2^) represents random additive genetic effects, where *K* is the kinship matrix; and *e ~ N*(0, *Iσ_e_*^2^) represents the residual error term.

Population structure was controlled by including the first five principal components derived from PCA as fixed covariates. Relatedness among individuals was accounted for using a kinship (*K*) matrix estimated within TASSEL. Each trait was analyzed separately using the same MLM framework.

### 2.7. Significance Threshold, Genomic Inflation Factor, and Identification of Independent Loci

To account for multiple testing, a Bonferroni correction was applied based on the total number of SNPs retained after quality control (*n* = 47,674), yielding a genome-wide significance threshold of

*p* < 1.05 × 10^−6^ (0.05/47,674)

A suggestive significance threshold was defined as

*p* < 2.10 × 10^−5^ (1/47,674)

No SNPs reached the genome-wide Bonferroni significance threshold in any of the seven traits analyzed, which is consistent with the moderate sample size (*n* = 401) and the complex polygenic architecture of growth traits. Therefore, SNPs with *p* < 1 × 10^−4^ were considered nominally associated loci and were used for candidate gene identification and biological interpretation.

Manhattan and quantile–quantile (QQ) plots were generated using the qqman package (v0.1.9) in R [18] to visualize association signals and evaluate model fit.

To assess residual population stratification or systematic bias, the genomic inflation factor (λ) was calculated for each trait using the median chi-square statistic:

(2) $$\lambda =\frac{median(\chi^{2}observed)}{\chi^{2}0.5,1}$$

The λ values ranged from 0.971 to 1.016 across the seven traits, indicating adequate control of population stratification and cryptic relatedness under the mixed linear model framework.

Independent loci were defined by grouping SNPs located within 500 kb of each other on the same chromosome, with the SNP exhibiting the smallest *p*-value within each region designated as the lead SNP.

### 2.8. Candidate Gene Identification

Candidate genes were identified based on the physical positions of lead SNPs using the *Ovis aries* reference genome annotation (NCBI RefSeq, OviAri4 assembly; accessed July 2026). Genes located within ±100 kb upstream or downstream of each lead SNP were considered potential candidate genes, reflecting the expected extent of linkage disequilibrium in sheep populations.

Transcript-level annotations were consolidated to retain unique gene symbols, thereby avoiding duplication resulting from multiple transcript isoforms. Across all seven traits, a total of 112 unique candidate genes were identified.

Genes associated with more than one trait were considered potential pleiotropic candidates. Shared genomic regions influencing multiple traits were identified by overlapping lead SNP positions across traits.

### 2.9. Candidate Gene Functional Annotation

To infer the potential biological relevance of the identified candidate genes, functional annotation was performed using publicly available genomic databases, including the National Center for Biotechnology Information (NCBI Gene), Ensembl Genome Browser, UniProt Knowledgebase, and the UCSC Genome Browser (accessed July 2026).

For each candidate gene, annotated gene descriptions, molecular functions, biological processes, and reported involvement in growth, skeletal development, metabolism, or regulatory pathways were retrieved from these databases. Given that functional annotation in sheep remains less comprehensive compared with model organisms, orthologous gene information from human (*Homo sapiens*), cattle (*Bos taurus*), mouse (*Mus musculus*), and rat (*Rattus norvegicus*) was used to infer conserved biological functions.

Orthologous relationships were determined using the Ensembl Genome Browser and the NCBI HomoloGene database (accessed July 2026). Functional interpretations were primarily based on experimentally validated evidence reported in mammalian systems.

To further evaluate the potential relevance of candidate genes to growth and developmental traits, a systematic literature search was conducted using PubMed (NCBI) and Google Scholar databases (accessed July 2026). Due to the moderate number of candidate genes and the exploratory nature of the associations, functional interpretation focused on literature-based biological relevance rather than formal pathway enrichment analysis.

### 2.10. QTL Overlap Analysis

To further evaluate the biological relevance of the identified loci, QTL overlap analysis was conducted using the Sheep QTL database (Animal QTLdb, Oar_v4 assembly; accessed July 2026) [18]. Genomic positions of the 44 independent significant SNPs were compared with reported QTL intervals based on physical coordinates. An overlap was defined when the SNP position fell within the start and end boundaries of a reported QTL region on the same chromosome.

## 3. Results

### 3.1. Genotype Harmonization and Quality Control

Following genome reference harmonization to Oar_v4 (OviAri4), genotype data from Qira Black sheep (50K SNP array), Kyrgyz sheep (WGS), and Dorset × Hu and Suffolk × Karakul crossbred sheep (WGS) were successfully integrated. After coordinate conversion and SNP identifier standardization, common loci corresponding to the 50K SNP panel were extracted from the WGS datasets.

The merged dataset initially contained 50,940 SNPs across 401 individuals. During dataset merging, multi-allelic conflicts were identified and removed, retaining only bi-allelic variants. Subsequent quality control filtering excluded 2974 SNPs with a genotype missing rate >5% and 292 SNPs with a minor allele frequency (MAF) <0.05. No individuals exceeded the 5% missingness threshold; therefore, no samples were excluded (Table 2).

The final high-quality dataset consisted of 401 individuals and 47,674 autosomal SNPs, with an overall genotyping rate of 99.8%.

### 3.2. Population Structure Analysis

**Figure 1:**
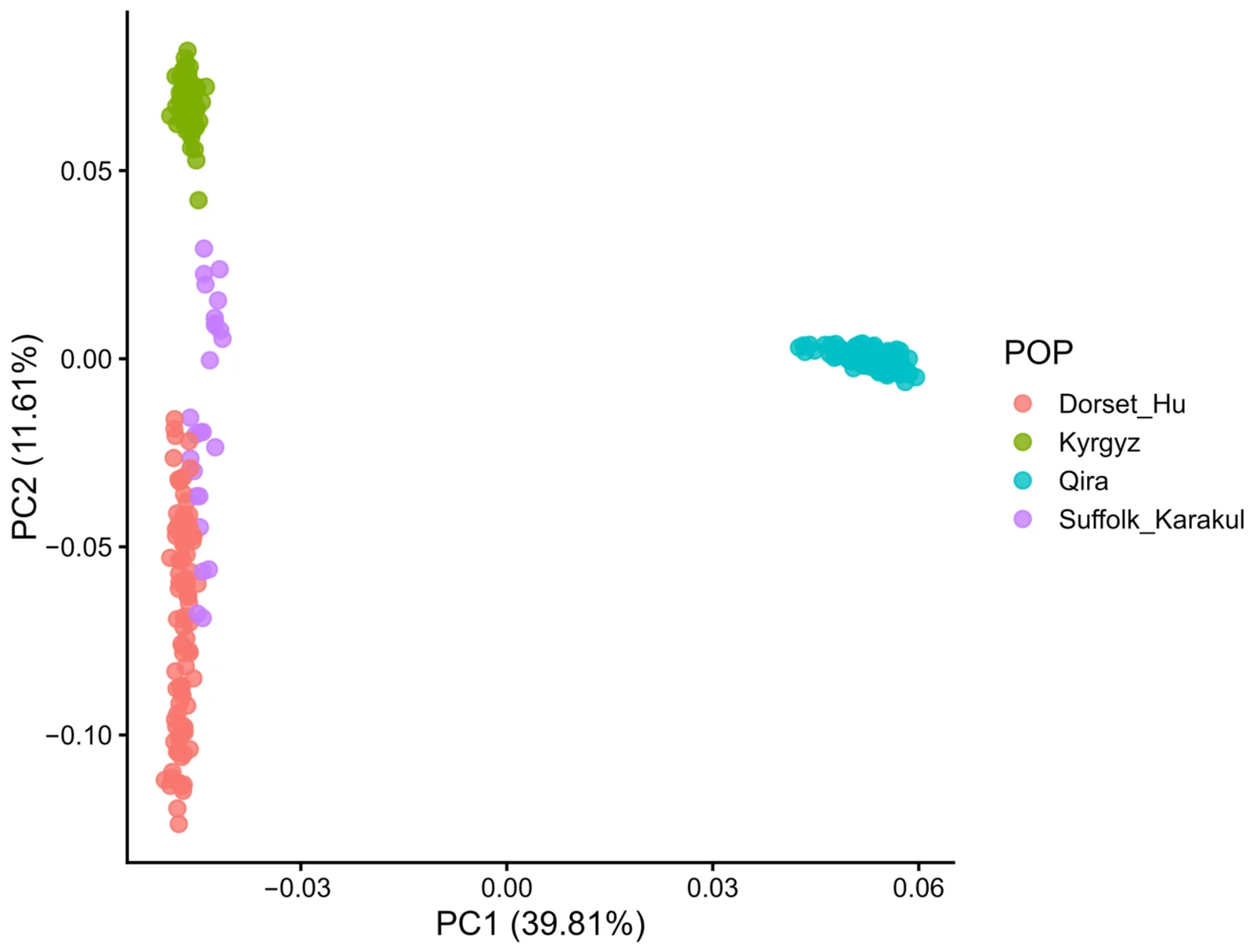
Principal component analysis based on LD-pruned SNPs revealed clear genetic differentiation among populations.

Principal component analysis (PCA) revealed clear genetic differentiation among Qira Black, Kyrgyz, Dorset × Hu crossbred, and Suffolk × Karakul crossbred sheep populations (Figure 1; Supplementary PCA_3D).

The first principal component (PC1) explained 39.81% of the total genetic variance and clearly separated Qira Black sheep from the other populations. The second principal component (PC2) accounted for 11.61% of the variance and distinguished the Kyrgyz population from the crossbred groups. The third principal component (PC3) explained 5.57% of the variance and primarily captured within-population variation, particularly among crossbred individuals.

In the three-dimensional space defined by PC1–PC3, crossbred animals were positioned intermediate between parental populations, consistent with their admixed genetic background. The first three principal components collectively explained 56.99% of the total genetic variation, indicating substantial population structure across the analyzed groups.

Qira Black sheep formed a compact and well-defined cluster, reflecting relative genetic homogeneity. In contrast, the crossbred populations—particularly Suffolk × Karakul—displayed broader dispersion, consistent with increased genomic heterogeneity resulting from crossbreeding.

### 3.3. Genome-Wide Association Analysis

Genome-wide association analyses were performed for seven growth and body conformation traits using a mixed linear model (MLM) incorporating breed, the first five principal components, and a kinship matrix to control for population structure and relatedness.

**Figure 2:**
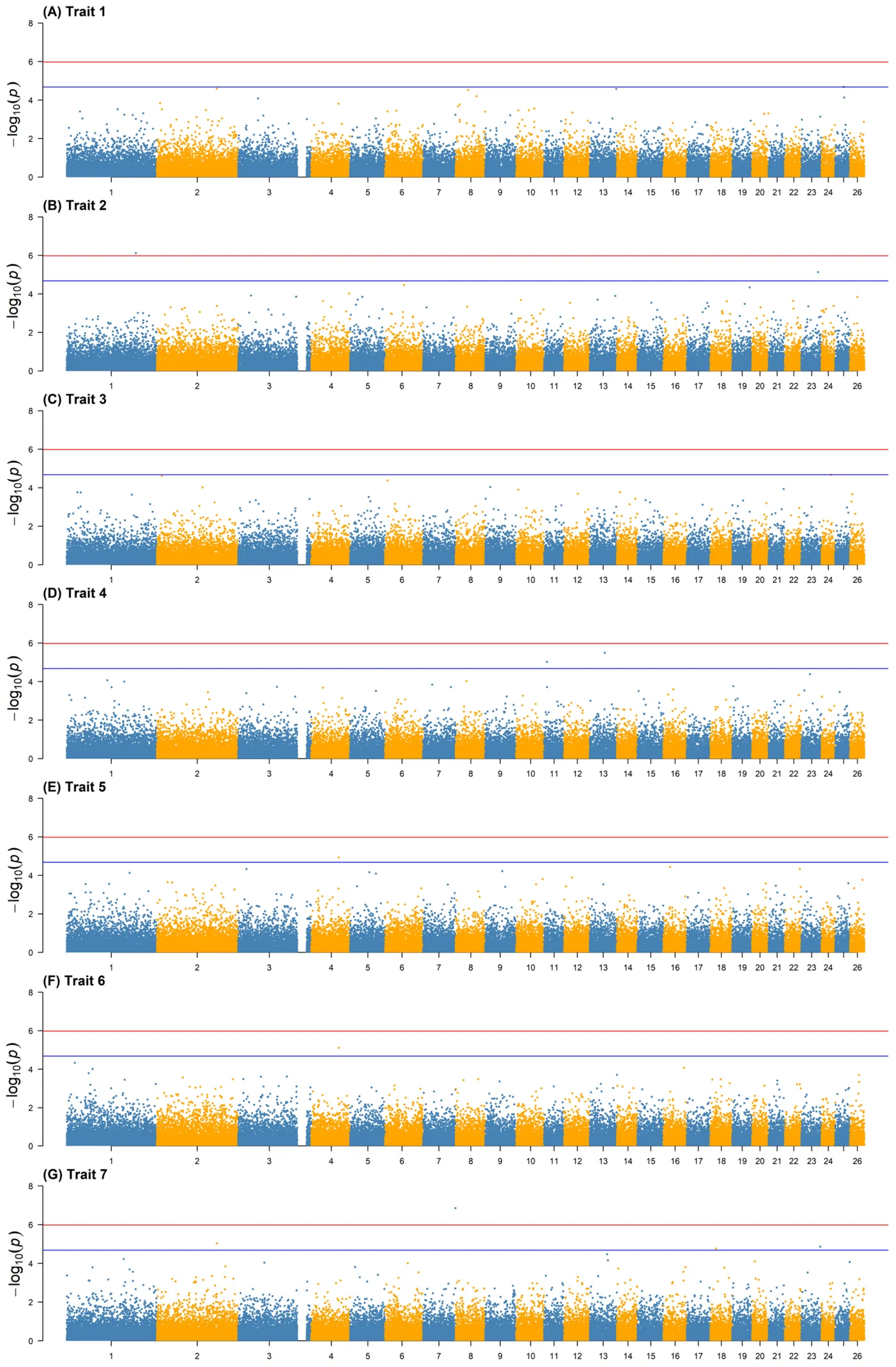
Manhattan plots showing genome-wide association results for seven growth and body conformation traits: (**A**) body height (BH), (**B**) body length (BL), (**C**) chest girth (ChG), (**D**) chest width (ChW), (**E**) cannon bone circumference (CBC), (**F**) hip height (HH), and (**G**) hip width (HW) in a multi-breed sheep population. The *x*-axis represents chromosomal position, and the *y*-axis represents the −log_10_(*p*) values of SNP associations. The red horizontal line indicates the Bonferroni-corrected genome-wide significance threshold (*p* < 1.05 × 10^−6^), and the blue line represents the nominal significance threshold (*p* < 1 × 10^−4^).

Manhattan plots for all traits are presented in Figure 2. No SNP reached the Bonferroni-corrected genome-wide significance threshold (*p* < 1.05 × 10^−6^). However, several loci exceeded the predefined nominal significance threshold (*p* < 1 × 10^−4^), indicating potential genomic regions associated with growth-related traits.

**Figure 3:**
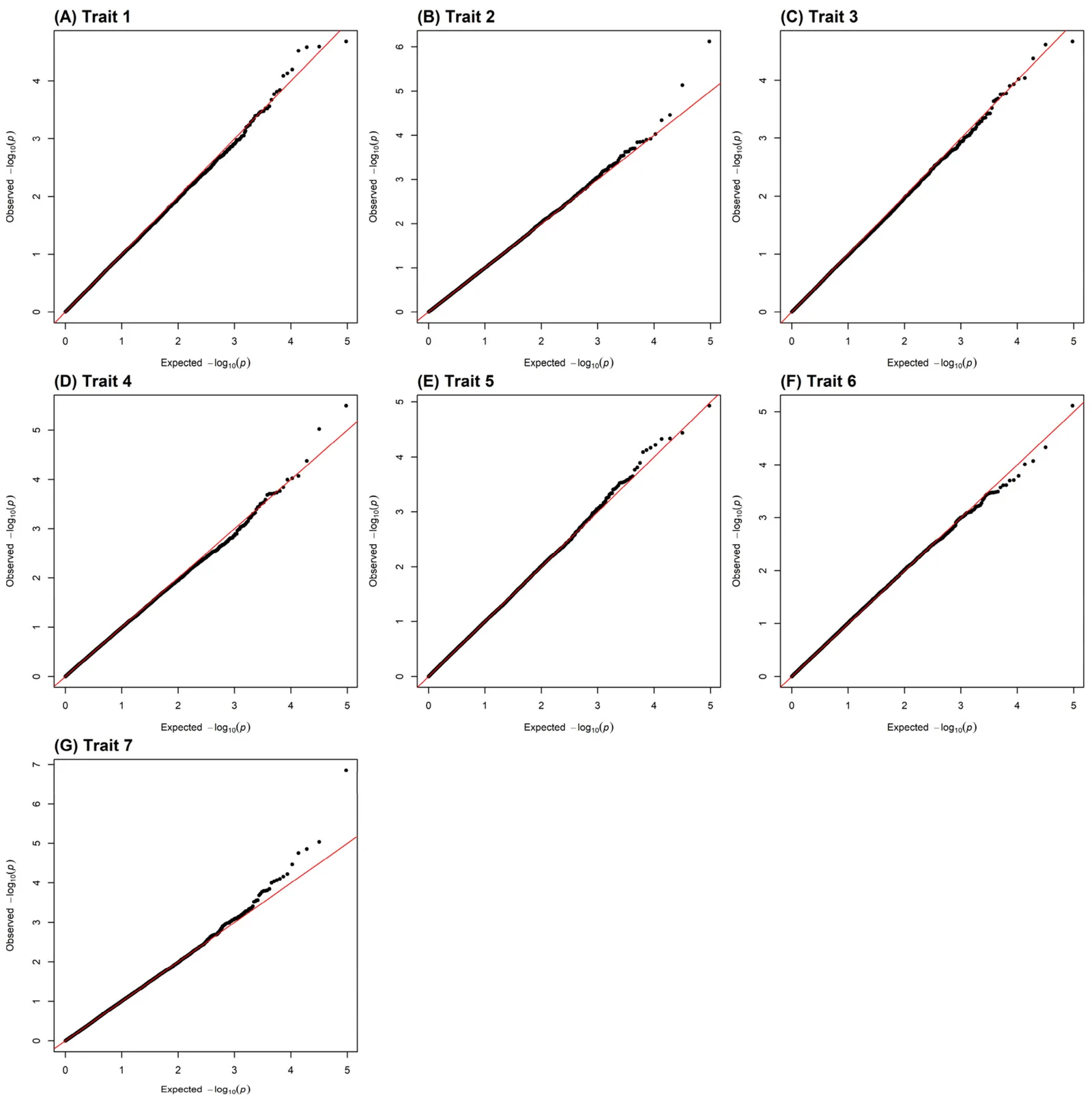
Quantile–quantile (QQ) plots of observed versus expected −log_10_(*p*) values for seven growth traits: (**A**) body height (BH), (**B**) body length (BL), (**C**) chest girth (ChG), (**D**) chest width (ChW), (**E**) cannon bone circumference (CBC), (**F**) hip height (HH), and (**G**) hip width (HW). The red diagonal line represents the null hypothesis of no association. Deviations in the upper tail indicate potential association signals. Genomic inflation factors (λ) ranged from 0.971 to 1.016, indicating effective control of population structure.

Quantile–quantile (QQ) plots (Figure 3) demonstrated close agreement between observed and expected *p*-value distributions across all traits, with deviation observed primarily in the extreme upper tail. The genomic inflation factor (λ) ranged from 0.971 to 1.016 (Supplementary Table S1), indicating effective control of population stratification and minimal residual inflation under the MLM framework.

These results indicate that the applied model appropriately accounted for population structure and relatedness while allowing detection of putative association signals.

### 3.4. Identification of Independent Associated Loci

To identify putative association signals, SNPs with *p* < 1 × 10^−4^ were considered nominally associated. SNPs located within 500 kb of each other on the same chromosome were grouped into single loci, and the SNP with the lowest *p*-value within each region was designated as the lead SNP.

Across the seven traits, a total of 44 independent loci were identified (Supplementary Table S2). The number of loci detected per trait ranged from four to ten, reflecting variability in the genetic architecture of the analyzed growth and body conformation traits.

The identified lead SNPs exhibited moderate association signals, and several loci contributed to phenotypic variation. Overall, these findings are consistent with a polygenic architecture underlying growth and body conformation traits in this multi-breed sheep population.

### 3.5. Candidate Gene Identification

Candidate genes were identified within a ±100 kb window surrounding each lead SNP based on the *Ovis aries* RefSeq annotation (OviAri4). Across all seven traits, 112 unique candidate genes were detected (Table 3; Supplementary Table S3). The number of genes per trait ranged from 5 to 36, reflecting local gene density and genomic architecture.

#### 3.5.1. Hip Height (HH)

Seven independent loci were identified across chromosomes 2, 3, 8, 13, and 25. The strongest association was observed at Chr25: 25,936,715 bp (*COL13A1*; −log_10_*p* = 4.68). Additional loci included Chr2: 183,645,748 bp (*STEAP3*, *DBI*, *TMEM37*); Chr3: 60,813,437 bp (*UXS1*, *ST6GAL2*); Chr8: 63,927,935 bp (*HECA*, *TXLNB*, *CITED2*); and Chr13: 80,730,709 bp (*LOC105608656*). These loci support the involvement of multiple genomic regions in regulating hip height.

#### 3.5.2. Hip Width (HW)

Five loci were detected on chromosomes 1, 4, 6, 19, and 23. The most significant association was observed at Chr1: 211,529,959 bp (*NLGN1*; −log_10_*p* = 6.12). Additional loci included Chr23: 51,905,023 bp (*DCC*; −log_10_*p* = 5.13); Chr6: 58,172,124 bp (*TMEM156*, *WDR19*); and Chr4: 116,121,440 bp (*DPP6*). These findings suggest multiple genomic regions potentially influencing skeletal width traits.

#### 3.5.3. Body Height (BH)

Five loci were detected across chromosomes 2, 6, 9, and 24. The strongest association was located at Chr24: 28,707,737 bp (*CALN1*; −log_10_*p* = 4.67). Two loci were identified on chromosome 2 at Chr2: 15,469,272 bp (*TRNAE-CUC*) and Chr2: 140,289,737 bp (*STK39* region). Additional associations included Chr9: 15,540,216 bp (*SLC45A4*, *DENND3*) and Chr6: 7899,382 bp (intergenic region).

#### 3.5.4. Body Length (BL)

Five loci were detected across chromosomes 1, 8, 11, 13, and 23. The strongest association was observed at Chr13: 45,839,084 bp (*DIP2C*, *ZMYND11*; −log_10_*p* = 5.49). Other loci included Chr11: 9405,361 bp (*PPM1F*, *TRIM37*) and Chr1: 124,064,451 bp (*GRIK1*).

#### 3.5.5. Chest Girth (ChG)

Eight loci were identified across chromosomes 1, 3, 4, 5, 9, 16, and 22. Notable candidate genes included *PPARGC1B* (Chr5), *SLC26A2* (Chr5), *ZFHX4* (Chr9), *RAB3C* (Chr16), and *CLRN3* and *PTPRE* (Chr22). These loci may contribute to metabolic and skeletal regulatory pathways relevant to thoracic development.

#### 3.5.6. Chest Width (ChW)

Four loci were detected across chromosomes 1, 4, and 16. Notable candidate genes included *COL11A1* (Chr1), *ELAVL4* (Chr1), and *TRNAS-GGA* (Chr4).

#### 3.5.7. Cannon Bone Circumference (CBC)

Ten loci were identified across chromosomes 1, 2, 3, 7, 13, 18, 20, 23, and 25. The strongest association in the study was detected at Chr7: 99,280,465 bp (*RPS6KA5*; −log_10_*p* = 6.85; *p* = 1.40 × 10^−7^). Additional loci included *STEAP3* (Chr2), *SOX18* (Chr13), and *NTRK3* (Chr18). The relatively large number of loci detected for CBC is consistent with a polygenic contribution to cortical bone morphology.

### 3.6. Shared Genomic Regions Across Traits

Two genomic regions were identified as being associated with multiple traits, suggesting potential pleiotropic effects (Supplementary Table S4).

A locus on chromosome 2 (lead SNP: 2:183,645,748 bp) was associated with both cannon bone circumference (CBC) and hip height (HH). This region harbors several candidate genes, including *STEAP3*, *DBI*, *TMEM37*, *LOC101111343*, and *LOC105609916*, suggesting a possible shared genetic contribution to skeletal growth dimensions.

Similarly, a locus on chromosome 4 (lead SNP: 4:84,387,399 bp) was associated with both chest girth (CG) and chest width (CW). The candidate interval includes *TRNAS-GGA*, indicating that this genomic region may contribute to correlated variation in thoracic growth traits.

The identification of shared loci across multiple growth traits is consistent with the presence of pleiotropic genomic regions contributing to morphological and developmental variation in this multi-breed sheep population. These overlapping signals may reflect coordinated genetic regulation of related skeletal and body conformation traits.

### 3.7. QTL Overlap with Significant SNPs

QTL overlap analysis revealed that four of the 44 independent significant SNPs were located within previously reported QTL intervals. Notably, the SNP at Chr4:84,387,399, associated with chest girth and chest width, overlapped with a body weight QTL region (Chr4:82,526,925–93,489,024). Given the well-documented phenotypic relationship between chest circumference traits and body weight, this overlap is consistent with the potential biological relevance of the identified association signal.

In addition, one locus overlapped with a tail length QTL interval, suggesting possible involvement in morphological development. The remaining overlapping loci corresponded to QTL traits unrelated to growth, indicating that not all detected associations coincide with previously reported growth-related regions.

## 4. Discussion

In this study, we performed a multi-breed GWAS for seven growth and body conformation traits in Qira Black, Kyrgyz, Dorset × Hu crossbred, and Suffolk × Karakul crossbred sheep. Growth traits are economically important and are widely recognized as being influenced by complex polygenic architectures. Previous GWAS in sheep have identified numerous loci associated with body weight and conformation traits, highlighting the genetic complexity underlying skeletal growth and development [7,19,20,21,22].

Using a mixed linear model controlling for population structure and kinship, we identified 44 independent loci encompassing 112 candidate genes. Although no locus reached Bonferroni significance, the detected signals and the presence of pleiotropic regions are consistent with a polygenic architecture for growth traits in this multi-breed population.

### 4.1. Skeletal Height and Limb-Related Traits (BH, HH, CBC)

Body height (BH), hip height (HH), and cannon bone circumference (CBC) reflect longitudinal bone growth and skeletal development. Several candidate genes identified in this study are functionally related to osteogenesis, cartilage biology, and growth signaling pathways.

For BH, loci near *STK39*, *SLC45A4*, *DENND3*, and *CALN1* were identified. *STK39* has been implicated in osteogenic differentiation and bone formation through NF-κB signaling [23]. *CALN1*, a calcium-binding protein, participates in calcium-mediated signaling pathways essential for osteoblast differentiation and skeletal growth [24]. These findings suggest that variation in calcium-dependent and kinase-mediated pathways may contribute to differences in body height.

For HH, genes including *UXS1*, *ST6GAL2*, *HECA*, *CITED2*, and *COL13A1* were detected. *UXS1* contributes to glycosaminoglycan biosynthesis and extracellular matrix formation, processes central to endochondral ossification [25]. *ST6GAL2* is regulated by TGF-β/SMAD signaling, a key pathway in chondrocyte differentiation [26]. *HECA* modulates mTOR signaling [27], while *CITED2* regulates osteoclast differentiation [28]. *COL13A1* participates in extracellular matrix organization and muscle-connective tissue interactions [29]. Collectively, these genes suggest coordinated regulation of cartilage formation, bone remodeling, and skeletal growth.

For CBC, the strongest signal in the study was detected near *RPS6KA5* (Chr7), a component of the MAPK pathway involved in cell proliferation and differentiation [30]. Additional genes, including *PLEKHH2*, *ZNF512B*, *DNAJC5*, and *PACSIN1*, are associated with cytoskeletal organization and developmental regulation, suggesting that cytoskeletal dynamics and mechanotransduction may influence cortical bone morphology [31,32,33,34].

### 4.2. Shared Genomic Regions and Pleiotropy

A locus on chromosome 2 (2:183,645,748) was associated with both HH and CBC. This region contains *STEAP3*, *DBI*, and *TMEM37*, genes involved in cartilage homeostasis and growth-related signaling pathways. *STEAP3* regulates MAPK and JAK/STAT signaling in cartilage [35], while *DBI* modulates autophagy and skeletal tissue maintenance [36]. *TMEM37* has been linked to intracellular Ca^2+^ regulation [37]. These shared associations suggest coordinated genetic influences on limb length and bone circumference.

Similarly, a chromosome 4 region containing *TRNAS-GGA* was associated with both chest girth and chest width. Previous evidence indicates that *TRNAS-GGA* is under selection and associated with body weight traits in Tibetan sheep [38], suggesting potential involvement in thoracic growth.

The identification of pleiotropic loci is consistent with shared developmental pathways underlying correlated skeletal traits.

### 4.3. Body Length and Frame Traits

Body length (BL) loci included *PPM1E* and *TRIM37*, both implicated in osteoblast differentiation and mesenchymal stem cell regulation [39,40]. These genes are linked to AMPK signaling and epigenetic regulation of osteogenesis, highlighting metabolic and transcriptional contributions to skeletal elongation.

For hip width (HW), loci near *DPP6*, *KLHL5*, *WDR19*, *XCR1*, and *DCC* were identified. *DPP6* has been associated with growth-related signaling pathways [41]. *XCR1* influences osteoblast differentiation via RUNX2-mediated regulation [42], and *DCC* participates in cartilage–bone interactions [43]. These findings suggest that pelvic morphology may be influenced by coordinated osteogenic and signaling mechanisms.

Chest girth (CG) loci included *OPA1*, *PPARGC1B*, *SLC26A2*, and *ZFHX4*, genes associated with mitochondrial function, energy metabolism, and endochondral ossification [44,45,46,47]. *SLC26A2* is particularly notable for its established role in cartilage matrix formation and skeletal dysplasia [46,48], supporting its relevance to thoracic development.

For chest width (CW), *COL11A1* was identified, a collagen gene critical for cartilage matrix organization and bone mineralization [49], consistent with structural determinants of rib cage morphology.

The overlap between the significant SNP at Chr4:84,387,399 and a previously reported body weight QTL is consistent with the biological relevance of this genomic region. Given the positive phenotypic correlation between chest circumference traits and body weight in sheep, this locus may contribute to overall body growth and skeletal development.

### 4.4. Polygenic Architecture and Study Limitations

No SNP reached the stringent Bonferroni-corrected genome-wide significance threshold. This likely reflects the moderate sample size and the highly polygenic architecture of growth and body conformation traits, which are typically influenced by numerous loci with small individual effects. In livestock populations, Bonferroni correction can be overly conservative due to linkage disequilibrium among markers, reducing the effective number of independent tests. Therefore, suggestive association signals may still provide biologically informative insights when supported by complementary evidence such as candidate gene annotation and QTL overlap analysis. Importantly, the genomic inflation factor (λ = 0.971–1.016) indicates effective control of population stratification and minimal residual inflation.

The multi-breed design of this study, while advantageous for increasing allelic diversity and broader applicability, introduces genetic heterogeneity and unequal sample sizes among populations, particularly within the crossbred groups. Such imbalance may reduce statistical power and complicate detection of breed-specific loci. Although principal component analysis (PCA) was incorporated into the association model to account for population structure, residual stratification effects cannot be completely excluded in multi-breed GWAS.

Furthermore, several significant loci were located in intergenic or poorly annotated regions, reflecting current limitations in ovine genome annotation. Although animals were within a similar age range (1–3 years), age was not included as a covariate in the model and may contribute minor residual phenotypic variation.

Future studies involving larger sample sizes, single-breed validation analyses, and functional experiments will be necessary to confirm causal variants and further elucidate the biological mechanisms underlying growth and body conformation traits.

## 5. Conclusions

This multi-breed genome-wide association study detected 44 independent loci associated with seven growth and body conformation traits across Qira Black, Kyrgyz, Dorset × Hu crossbred, and Suffolk × Karakul crossbred sheep. Although no variant surpassed the stringent Bonferroni-corrected threshold, several biologically plausible candidate genes potentially involved in skeletal development, cartilage biology, and metabolic regulation were identified, including *RPS6KA5*, *STEAP3*, *SLC26A2*, *COL11A1*, and *PPARGC1B*. The observation of shared genomic regions across traits suggests possible pleiotropic genetic influences on correlated body frame characteristics.

Overall, the findings are consistent with a polygenic architecture underlying growth and body conformation traits in sheep. While further validation in larger and independent populations is required, this study provides preliminary insights into genomic regions that may contribute to growth-related phenotypes and offers a reference framework for future genetic and functional investigations in indigenous and crossbred sheep populations.

## Figures and Tables

**Table 1:** Descriptive statistics (mean ± SD) of seven body conformation traits across four sheep populations.

Trait	Qira Black (*n* = 186)	Kyrgyz (*n* = 100)	Dorset × Hu (*n* = 90)	Suffolk × Karakul (*n* = 25)
Hip Height	62.48 ± 3.77	68.88 ± 3.61	63.33 ± 3.83	67.72 ± 3.98
Hip Width	11.00 ± 1.91	11.91 ± 1.10	15.43 ± 2.90	16.72 ± 1.98
Body Height	65.18 ± 4.35	48.89 ± 4.56	62.73 ± 3.65	67.34 ± 4.13
Body Length	63.15 ± 5.63	63.92 ± 5.07	71.42 ± 4.53	75.48 ± 5.74
Chest Girth	83.62 ± 7.61	93.93 ± 8.13	83.39 ± 5.33	95.52 ± 8.06
Chest Width	21.81 ± 2.55	29.06 ± 2.60	19.91 ± 2.53	25.49 ± 3.13
Cannon Bone Circumference	7.64 ± 0.72	8.95 ± 0.58	7.89 ± 0.58	9.08 ± 0.84

**Table 2:** Distribution of SNPs before and after quality control.

CHR	SNPs BEFORE QC	SNPs AFTER QC	LENGTH (BP)	AVG DIST BEFORE (KB)	AVG DIST AFTER (KB)
1	5673	5351	275,387,639	48.54356	51.46471
2	5162	4903	248,856,506	48.20932	50.75597
3	4249	3953	223,909,211	52.69692	56.64286
4	2475	2316	119,170,624	48.14975	51.45536
5	2287	2175	106,803,928	46.70045	49.10525
6	2807	2633	116,838,627	41.62402	44.37472
7	2173	2057	99,977,231	46.00885	48.60342
8	1939	1832	90,508,444	46.67790	49.40417
9	2005	1903	94,534,672	47.14946	49.67665
10	1792	1646	86,345,120	48.18366	52.45755
11	1279	1174	61,956,862	48.44164	52.77416
12	1709	1606	79,016,807	46.23570	49.20100
13	1701	1578	82,928,768	48.75295	52.55308
14	1342	1219	62,544,241	46.60525	51.30783
15	1694	1590	80,663,623	47.61725	50.73184
16	1509	1408	71,655,186	47.48521	50.89147
17	1426	1325	72,084,885	50.55041	54.40369
18	1593	1444	68,390,719	42.93203	47.36199
19	1185	1122	60,365,139	50.94105	53.80137
20	1155	1078	50,816,866	43.99729	47.13995
21	950	894	49,944,548	52.57321	55.86638
22	1089	1026	50,770,794	46.62148	49.48420
23	1117	1033	62,251,113	55.73063	60.26245
24	787	707	41,968,799	53.32757	59.36181
25	989	904	45,193,605	45.69626	49.99293
26	853	797	43,975,533	51.55397	55.17633

**Table 3:** Chromosome-wise significant SNPs associated with growth and meat production traits.

Traits	Chr	Position	Beta (Add)	−log_10_(*p*)	Nearest Gene	*p*-Value
BH	2	15469272	1.18052	4.62	*TRNAE-CUC*	2.42 × 10^−5^
BH	2	140289737	0.1945	4.02	*STK39*, *LOC105609277*	9.55 × 10^−5^
BH	6	7899382	−1.8581	4.38	*-*	4.20 × 10^−5^
BH	9	15540216	−1.8824	4.04	*SLC45A4*, *DENND3*	9.16 × 10^−5^
BH	24	28707737	−1.3944	4.67	*CALN1*	2.14 × 10^−5^
BL	1	124064451	1.73526	4.07	*GRIK1*	8.49 × 10^−5^
BL	8	33138639	−3.3348	4.02	*LOC105609889*, *TRNAM-CAU*	9.49 × 10^−5^
BL	11	9405361	0.37603	5.02	*PPM1E*, *TRIM37*	9.49 × 10^−6^
BL	13	45839084	5.48519	5.49	*TRNAC-GCA*, *DIP2C*, *ZMYND11*	3.21 × 10^−6^
BL	23	26780948	1.95098	4.38	*-*	4.18 × 10^−5^
ChG	1	192018252	1.1291	4.12	*OPA1*, *ATP13A4*	7.52 × 10^−5^
ChG	3	25571247	2.9336	4.33	*VSNL1*, *SMC6*, *TRNAW-CCA*, *GEN1*	4.70 × 10^−5^
ChG	4	84387399	−2.1502	4.93	*TRNAS-GGA*	1.17 × 10^−5^
ChG	5	58665392	−1.23	4.17	*PPARGC1B*, *PDE6A*, *SLC26A2*, *TIGD6*, *HMGXB3*	6.81 × 10^−5^
ChG	5	78853492	0.48283	4.09	*SSBP2*	8.18 × 10^−5^
ChG	9	52489049	−2.4119	4.22	*LOC105611157*, *LOC105611154*, *LOC106991349*, *ZFHX4*	6.04 × 10^−5^
ChG	16	20773348	3.29843	4.44	*RAB3C*, *GAPT*	3.63 × 10^−5^
ChG	22	46211590	4.53616	4.34	*CLRN3*, *PTPRE*	4.61 × 10^−5^
ChW	1	24500579	0.35747	4.33	*ELAVL4*	4.72 × 10^−5^
ChW	1	79249563	1.37116	4.01	*COL11A1*	9.84 × 10^−5^
ChW	4	84387399	−0.45892	5.11	*TRNAS-GGA*	7.73 × 10^−6^
ChW	16	63359322	1.00698	4.07	*LOC105602631*, *LOC101122889*	8.49 × 10^−5^
CBC	1	174053426	0.31043	4.22	*LOC101113722*, *PVRL3*	5.98 × 10^−5^
CBC	2	183645748	0.13666	5.03	*STEAP3*, *LOC101111343*, *LOC105609916*, *DBI*, *TMEM37*	9.27 × 10^−6^
CBC	3	80440526	−0.18954	4.04	*PLEKHH2*, *C1GALT1C1L*, *LOC106990749*, *THADA*, *LOC101114943*, *LOC105614223*	9.15 × 10^−5^
CBC	7	99280465	0.27744	6.85	*RPS6KA5*	1.40 × 10^−7^
CBC	13	53151500	0.01445	4.47	*TCEA2*, *SOX18*, *LOC105616788*, *PRPF6*, *SAMD10*, *ZNF512B*, *UCKL1*, *DNAJC5*, *TPD52L2*, *ABHD16B*, *ZBTB46*	3.38 × 10^−5^
CBC	13	55465620	0.09754	4.16	*-*	6.97 × 10^−5^
CBC	18	18327839	0.09671	4.76	*NTRK3*	1.75 × 10^−5^
CBC	20	8642394	0.26678	4.10	*RPS10*, *PACSIN1*, *SPDEF*, *LOC105603662*, *C20H6orf106*	7.97 × 10^−5^
CBC	23	58211662	0.17595	4.86	*LOC105604562*, *ZNF532*, *LOC106991877*, *LOC101116529*, *LOC105604563*	1.38 × 10^−5^
CBC	25	44284433	0.01821	4.07	*-*	8.59 × 10^−5^
HH	2	183645748	0.23156	4.59	*STEAP3*, *LOC101111343*, *LOC105609916*, *DBI*, *TMEM37*	2.55 × 10^−5^
HH	3	60813437	−1.0446	4.09	*UXS1*, *LOC101114763*, *LOC101115015*, *LOC101108040*, *ST6GAL2*	8.17 × 10^−5^
HH	8	38395361	1.216	4.52	*HECA*, *TXLNB*, *CITED2*, *LOC105608886*	3.01 × 10^−5^
HH	8	63927935	1.15734	4.20	*-*	6.31 × 10^−5^
HH	13	80730709	−1.614	4.58	*LOC105608656*, *LOC105608658*	2.61 × 10^−5^
HH	25	25936715	1.83013	4.68	*COL13A1*, *LOC101108627*, *LOC105604970*, *AIFM2*	2.07 × 10^−5^
HH	25	27058660	−1.1679	4.13	*-*	7.38 × 10^−5^
HW	1	211529959	0.17229	6.12	*NLGN1*, *LOC105603240*, *LOC105603230*	7.57 × 10^−7^
HW	4	116121440	0.6221	4.03	*DPP6*, *LOC101106163*	9.43 × 10^−5^
HW	6	58172124	0.71105	4.46	*TMEM156*, *KLHL5*, *LOC101109179*, *WDR19*	3.47 × 10^−5^
HW	19	53215029	1.00108	4.34	*LOC101103766*, *LOC101117971*, *LOC105603561*, *XCR1*, *FYCO1*, *CXCR6*	4.56 × 10^−5^
HW	23	51905023	0.88262	5.13	*DCC*	7.35 × 10^−6^

## Data Availability

The data presented in this study are available from the corresponding author upon reasonable request. The data are not publicly available due to ownership and commercial considerations.

## Supplementary Materials

The following supporting information can be downloaded at https://www.mdpi.com/article/10.3390/ani16162605/s1. Table S1: The genomic inflation factor; Table S2: A total of independent SNP loci across the seven traits; Table S3: The unique candidate genes across all seven traits; Table S4: Shared genomic regions across traits.
